# Post‐traumatic hypopituitarism: report of a child case

**DOI:** 10.1002/ams2.220

**Published:** 2016-05-16

**Authors:** Makoto Aoki, Shuichi Hagiwara, Masato Murata, Minoru Kaneko, Masahiko Kanbe, Jun Nakajima, Yusuke Sawada, Yoshio Ohyama, Jun'ichi Tamura, Kiyohiro Oshima

**Affiliations:** ^1^ Department of Emergency Medicine Gunma University Graduate School of Medicine Maebashi Gunma Japan; ^2^ Department of General Medicine Gunma University Graduate School of Medicine Maebashi Gunma Japan; ^3^ Emergency and General Medical Center Gunma University Hospital Maebashi Gunma Japan

**Keywords:** Child, diabetes insipidus, head trauma, hypopituitarism

## Abstract

**Case:**

We report a case of post‐traumatic hypopituitarism in a 9‐year‐old boy who was injured in a car accident.

**Outcome:**

Post‐traumatic hypopituitarism might be caused by moderate to severe head trauma, and while this possibility has recently drawn attention in adults, few reports are available regarding children. Our patient experienced head and facial injury, resulting in post‐traumatic hypopituitarism. Six hours after injury he suffered from diabetes insipidus and hormone replacement therapy was started. On day 12 he underwent facial fracture reduction under general anesthesia. On day 24 he was discharged from the hospital. One year after the injury, secretory function and water dehydration tests suggested the possibility of post‐traumatic hypopituitarism.

**Conclusion:**

We experienced a child case of post‐traumatic hypopituitarism. Emergency physicians should pay attention to the possibility of post‐traumatic hypopituitarism in cases of traumatic brain injury.

## Introduction

The importance of hypopituitarism as a consequence of traumatic brain injury (TBI) was recently highlighted in adult patients; however, few systematic studies on post‐traumatic hypopituitarism exist in children.^1^ We describe the case of a child who had post‐traumatic hypopituitarism following TBI caused by a traffic accident. The patient gave written informed consent for publication of this case report and all accompanying images.

## Case

A 9‐year‐old boy riding in the back seat of a car was injured when the car collided with a motorcycle. He was transferred to our emergency room by helicopter. On arrival, he exhibited disturbed consciousness and his Glasgow Coma Scale score was 10 (eye, 1; verbal, 4; movement, 5). His height and body weight were 126 cm and 45.9 kg, respectively. Although he was heavy for his age, he had no past medical history. His vital signs included blood pressure of 116/67 mmHg, heart rate of 64 b.p.m., body temperature of 37.0°C, respiratory rate of 24/min, and SpO_2_ of 100% (face mask with reservoir, O_2_ 10 L/min). He had a small nosebleed. Swelling and contusions were observed from the right inner orbit to the nose, and there were other contusions and many bits of glass on his face.

**Figure 1:**
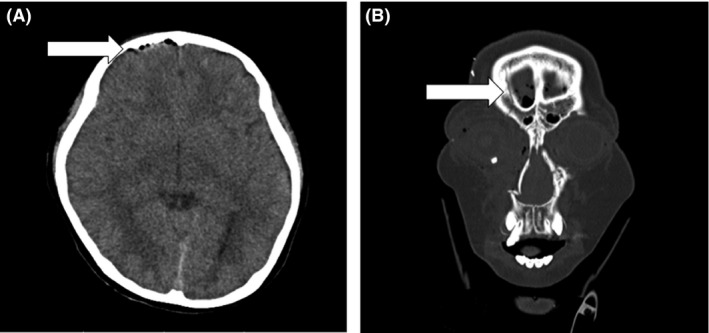
Computed tomography of a 9‐year‐old boy who was injured in a car accident, carried out on arrival at hospital. A, Pneumocephalus inside the frontal cranial bone (arrow). B, Fracture of the frontal cranial bone (arrow).

**Figure 2:**
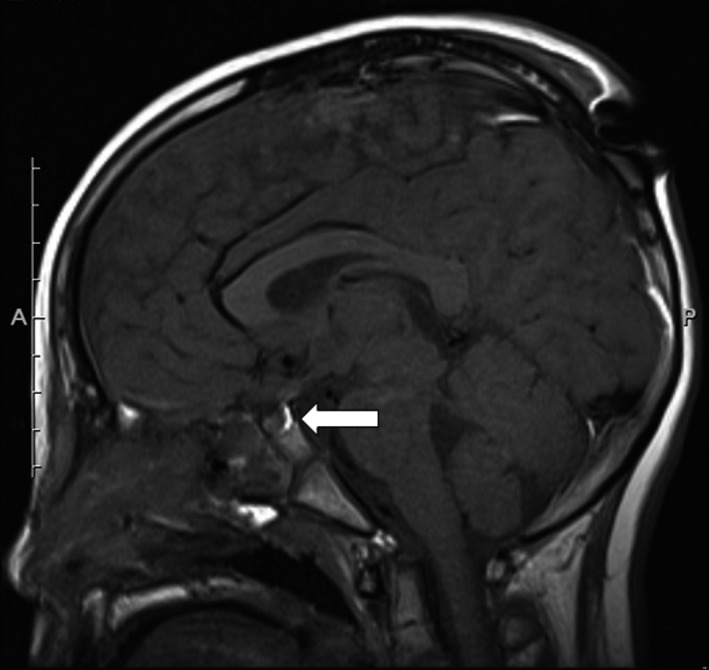
Head magnetic resonance imaging of a 9‐year‐old boy who was injured in a car accident. The T1‐weighted image shows the normal appearance of a high intensity signal in the posterior pituitary lobe (arrow).

Computed tomography (CT) carried out on arrival showed that there was a subdural hematoma at the cerebral falx, pneumocephalus, and fractures of the front cranial bone, ethmoid bone, the orbital floor, and nasal bone (Fig. 1). A second CT carried out 5 h after the first revealed that the subdural hematoma had not expanded, and his consciousness had gradually recovered; 8 h after injury his Glasgow Coma Scale score was 15. He was moved to the intensive care unit. Six hours after injury, his urine volume suddenly increased (>10 mL/kg/h) and his urine‐specific gravity was remarkably low (1.001). We diagnosed him with post‐traumatic diabetes insipidus and started i.v. treatment with continuous vasopressin. One hour following the administration of vasopressin, his urine volume was controlled at approximately 1.5 mL/kg/h and urine‐specific gravity recovered to within normal limits. Laboratory studies on day 3 revealed the following hormone concentrations: growth hormone (GH), 0.50 ng/mL; follicle‐stimulating hormone, 0.3 mIU/mL; luteinizing hormone, 0.07 mIU/mL; adrenocorticotropic hormone, 13.4 pg/mL; and thyroid stimulating hormone (TSH), 1.13 μIU/mL. Nearly all findings were within normal limits for a prepubertal boy. Because vasopressin was effective, we concluded that the central diabetes insipidus was based on either dysfunction of the hypothalamic neurons secreting antidiuretic hormone or dysfunction of the posterior pituitary gland causing post‐traumatic diabetes insipidus. Laboratory data, including pituitary hormones, were followed up (Table 1). T1‐weighted magnetic resonance imaging performed on day 6 showed the normal appearance of a high intensity signal in the posterior pituitary lobe and of an isointense signal in the anterior pituitary lobe (Fig. 2). Hypothalamus was of normal appearance. On day 12, the patient underwent an operation for his facial fractures under general anesthesia. The postoperative course was uneventful. The dose of vasopressin was tapered based on regular measurements of serum sodium concentration and urine volume, and its administration route was changed from i.v. to nasal on day 18. A pituitary stimulation test carried out on day 24 revealed decreased levels of peak GH (3.40 ng/mL) and peak TSH (5.33 μIU/mL); thus, a repeat pituitary stimulation test was scheduled for 1 year after the injury. He was discharged from our hospital on the same day and was scheduled for a routine follow‐up by pediatric physicians. His basal hormone levels were maintained and only vasopressin was given continuously. One year after the injury, secretary function tests (pituitary, thyroid, reproductive, and adrenal glands) and a water dehydration test were carried out (Tables 2 and 3). Hormone provocative tests were undertaken using 27 g arginine, 100 μg gonadotropin‐releasing hormone (GnRH), 500 μg thyrotropin‐releasing hormone (TRH), and 80 μg corticotropin‐releasing hormone (CRH). Secretion of GH was also low; however, his height was within the normal range. The peak luteinizing hormone (9.8 mIU/mL) was higher than the peak follicle stimulating hormone (6.3 mIU/mL), so there was a possibility of secretory deficiency in puberty. His fT4 was in the normal range; however, TSH secretion was low, so he was considered to have central hypothyroidism. The peak adrenocorticotropic hormone and cortisol levels induced by the CRH tests were 103.8 pg/mL and 35.6 μg/dL, respectively, indicating normal function of the CRH– adrenocorticotropic hormone–cortisol axis. In water dehydration tests, the urine osmolarity remained low and the patient body weight was decreased by approximately 3% compared with that of the previous day. Urine osmolarity was increased after the injection of vasopressin. Consequently, an anterior pituitary function test revealed the possibility of partial hypopituitarism, and water dehydration tests revealed central diabetes insipidus. Treatment with levothyroxine sodium hydrate was started 12 months following the injury, and vasopressin was still required at least 15 months following the injury. Monthly follow‐ups have been carried out, and secretary function tests were scheduled for every 2 years.

**Table 1:** Endocrine concentrations 3 days, 3 months, and 9 months after traumatic brain injury in a 9‐year‐old boy

Hormone concentrations	3 days	3 months	9 months
GH, ng/mL (0.0–2.0)†	0.50	1.60	ND
PRL, ng/mL (3.58–12.7)	9.60	15.8	ND
FSH, mIU/mL (1.4–13.0)	0.30	1.70	2.00
LH, mIU/mL (0.79–5.72)	0.07	0.50	0.60
FT_4_, ng/dL (0.89–1.53)	1.05	1.29	1.20
FT_3_, pg/mL (1.9–4.9)	2.32	ND	ND
Morning ACTH, pg/mL (7.2–63.3)	13.4	49.1	44.8
Morning cortisol, μg/dL (4.0–19.3)	17.4	19.9	12.2
IGF‐1, ng/mL (84–350‡)	140	ND	204
TSH, μIU/mL (0.35–4.94)	1.13	2.09	2.50

†Normal ranges are given in parentheses in the first column. ‡Normal range of insulin‐like growth factor‐1 (IGF‐1) is age‐ and gender‐normalized. ACTH, adrenocorticotropic hormone; FSH, follicle stimulating hormone; FT_3_, free triiodothyronine; FT_4_, free thyroxine; GH, growth hormone; LH, luteinizing hormone; ND, not determined; PRL, prolactin; TSH, thyroid stimulating hormone.

**Table 2:** Responses of pituitary and adrenal hormones to i.v. injection of arginine (27 g), gonadotropin‐releasing hormone (100 μg), corticotropin‐releasing hormone (80 μg), and thyrotropin‐releasing hormone (500 μg) in a 10‐year‐old boy, 1 year after traumatic brain injury

Time, min	0	30	60	90	120
GH, ng/mL (0.0–2.0)†	<0.1	1.90	0.80	0.20	2.10
PRL, ng/mL (3.58–12.7)	9.9	21.4	13.3	8.8	6.7
FSH, mIU/mL (1.4–13.0)	2.0	4.3	5.8	6.3	6.0
LH, mIU/mL (0.79–5.72)	0.3	8.0	9.8	7.9	7.3
FT_4_, ng/dL (0.89–1.53)	1.20	ND	ND	ND	1.40
ACTH, pg/mL (7.2–63.3)	73.5	103.8	74.7	36.6	ND
Cortisol, μg/dL (4.0–19.3)	13.8	30.6	35.6	29.1	22.0
TSH, μIU/mL (0.35–4.94)	1.49	5.81	3.43	2.56	2.09

^†^Normal ranges are given in parentheses in the first column. ACTH, adrenocorticotropic hormone; FSH, follicle stimulating hormone; FT_4_, free thyroxine; GH, growth hormone; LH, luteinizing hormone; ND, not determined; PRL, prolactin; TSH, thyroid stimulating hormone.

**Table 3:** Water dehydration test in a 10‐year‐old boy, 1 year after traumatic brain injury

Time, h	Urine osmolarity, mOsm/kg
Baseline	338
1 h	271
2 h	323
0.5 h post‐injection vasopressin	420
1 h post‐injection vasopressin	644
1.5 h post‐injection vasopressin	696

## Discussion

Traumatic brain injury is a worldwide health problem because it is a major cause of disability and death as well as a cause of neuroendocrine dysfunction. Post‐traumatic hypopituitarism occurs with high frequency following severe or moderate TBI, and undiagnosed and untreated post‐traumatic hypopituitarism may contribute significantly to the morbidity associated with TBI.^2^ There are many reports on the incidence of hypopituitarism following TBI in adults, which show that some degree of pituitary dysfunction was found in 25% of adult patients after TBI.^3^ However, few systematic studies on post‐traumatic hypopituitarism exist in children;^1^ only a few case reports or small series of pediatric patients with hypopituitarism following TBI have been published.^4^, ^5^ Acerini *et al*.^6^ reported that hypopituitarism generally occurred after severe TBI and was often associated not only with loss of consciousness, subdural hematoma, or skull fracture, but also following relatively mild head injury without loss of consciousness.

Central diabetes insipidus occurs in 1 in 5 to 1 in 3 patients with post‐traumatic hypopituitarism.^7^ Agha *et al*. reported that 26% of patients with TBI developed central diabetes insipidus in the acute phase (the duration of in‐hospital stay)^8^ and that central diabetes insipidus occurred in 6.9% of long‐term survivors following TBI.^9^ Post‐traumatic central diabetes insipidus is frequently transient,^6^, ^10^ although spontaneous recovery from anterior pituitary hormone deficiency is rare.^10^ In one series, only 3 of 19 children (aged 4 months–15 years) with acute central diabetes insipidus secondary to severe brain injury survived the acute attack of central diabetes insipidus.^8^ Therefore, the occurrence of acute onset central diabetes insipidus may be a poor prognostic factor for survival in patients with TBI.

In this case, we could have diagnosed the central diabetes insipidus because the patient had developed the uncomplicated symptom of polyuria. Post‐traumatic hypopituitarism patients might show unspecific conditions, such as slow recovery, lack of muscle power, and marked lethargy and brain damage often presents with signs and symptoms that can easily mask the symptoms of hypopituitarism. Thus, emergency physicians who have little awareness of hypopituitarism could misdiagnose the condition.^11^ In 20% of patients with post‐traumatic hypopituitarism, pituitary imaging showed no abnormality of the sella region,^12^ therefore it is possible that our patient had hypopituitarism without pituitary imaging abnormalities.

There is no definite recommendation that routine endocrine evaluation should be carried out following TBI.^13^, ^14^ Auble *et al*. recommended that any child with a history of TBI be followed closely for growth velocity and pubertal changes. If growth velocity is slow, prolactin level and a full endocrine evaluation should be undertaken.

## Conclusion

In our case, we diagnosed post‐traumatic central diabetes insipidus based on the presence of polyuria; however, it is sometimes difficult to make this diagnosis if the patient has no remarkable symptoms. Emergency physicians should be aware of post‐traumatic hypopituitarism, especially in children. Any child with moderate to severe TBI is recommended to be followed up closely by a pediatrician for growth velocity and pubertal changes.

## Conflict of interest

None.
